# Psychological Antecedents and Consequences of Social Integration Based on Self-Disclosure in Virtual Communities: Empirical Evidence From Sina Microblog

**DOI:** 10.3389/fpsyg.2022.829327

**Published:** 2022-02-16

**Authors:** Yixin Zhang, Zhichao Cheng, Yue Pan, Yiwen Xu

**Affiliations:** ^1^School of Economic and Management, Beihang University, Beijing, China; ^2^School of Mechanical Engineering and Automation, Beihang University, Beijing, China

**Keywords:** virtual community, self-disclosure, social integration, intimacy, cognitive communion, psychological well-being

## Abstract

**Introduction:**

With the normalization of COVID-19 prevention and control, a large number of intergenerational audiences with different cognition preferences and value orientations have started to pour into non-acquaintance virtual communities (VCs) to address their social needs by disclosing their own thoughts, feelings and experiences toward certain topics. To avoid the negative impacts of self-disclosure, this study introduced the concept of social integration into cyber society among non-acquaintance VCs, such as the topic-based VCs. Our theoretical model considers both the psychological antecedents and consequences of VC audiences’ social integration and our findings have implications for public online (and even offline) social life. Moreover, this research could play a guiding role in improving VC audiences’ social integration status in future online learning and telecommuting scenarios.

**Method:**

To assess the theoretical model constructed in this manuscript, we conducted an online survey in two different topic-based VCs among Microblog and yielded 472 useable responses from intergenerational audiences, among which 28.81% were born before 1985, 26.67% were born from 1985 to 1995, and 48.52% were born after 1995. Our sample consisted of 208 individuals from Health Regimen VC and 264 individuals from Star Chasing VC, 200 (42.37%) were men and 272 (57.63%) were women.

**Results:**

Our structural equation model (SEM) indicated that individuals’ self-disclosure in topic-based VCs might not directly guide them to acquire social integration. However, intimacy and cognitive communion derived from VC audiences’ self-disclosure might not only enhance their social integration, but also improve their psychological well-being. In addition, VC audiences’ social integration mediated the relationship between intimacy and psychological well-being, and the relationship between cognitive communion and psychological well-being. Moreover, VC audiences’ intimacy was found to have a direct influence on their cognitive communion.

**Conclusion:**

In the context of topic-based VCs, audiences’ self-disclosure could significantly foster their intimacy and cognitive communion with others, and both intimacy and cognitive communion are conductive to VC audiences’ social integration. Thus, audiences in topic-based VCs who wish to improve their psychological well-being need to disclose themselves and build corresponding psychological foundations (i.e., intimacy and cognitive communion) to enhance their social integration. Meanwhile, topic-based VCs should pay attention to the cultivation of intimacy and cognitive communion among audiences while encouraging them to reveal themselves.

## Introduction

Under the circumstances of normalized isolation and prevention in post COVID-19, public social activities have been severely restricted, and public mental health needs to be improved urgently (Barzilay et al., 2020; Cruyt et al., 2021). Thus, non-acquaintance virtual communities (VCs) on Social Networking Sites (SNS) have started to accept enormous intergenerational audiences with different cognitive preferences and value orientations. A report from Statista (2021) shows that the frequency of social media usage by SNS audiences worldwide aged from 16 to 64 increased by 44% in March, 2020. As the most influential SNS in China, the user composition of Sina Microblog also changed in 2020. Sina Weibo Data Center (2021) indicated that 66% of its users were still relatively young (aged 20–40) in 2020, but the proportion of users in this age group once reached 80% in 2018. Meanwhile, the share of Sina Microblog users aged 40 and over in 2020 also increased by approximately 4% compared to 2018. Obviously, real society in the physical world has gradually been replaced by cyber society in the virtual world. Non-acquaintance VCs among SNSs have provided great public places for individuals to disclose themselves (including their thoughts, feelings, and even experiences) regarding certain topics (Vromen et al., 2015; Hu et al., 2016; Peng et al., 2018), and supertopic communities among Sina Microblog are typical representatives of topic VCs. According to social cognitive theory, people usually show different behaviors in different environments, and these behaviors can encourage them to choose, construct and strengthen the environments in which they study, live, and work (Bandura, 2001). Therefore, the virtual environment provided by topic-based VCs would greatly diversify individuals’ participation form (Chiang and Hsia, 2015; Hu et al., 2016). As self-disclosure can effectively facilitate individuals’ interactions, service customization, and digital content generation (Chen and Sharma, 2013), it has become the most common type of participation in topic-based VCs. Current studies related to VC audiences’ self-disclosure mainly focus on its group-level motivations (such as social support and social relationships) (Chen and Li, 2017; Lee et al., 2021) and its group-level consequences (such as commitment and stickiness) (Lin et al., 2021), but few studies have comprehensively analyzed the individual-level influences of VC audiences’ self-disclosure, such as psychological perceptions.

Generally, topic-based VCs (also called interest-based VCs) can help individuals who do not have strong ties but are drawn on to the same topic to easily gather together and interact with each other (Shen et al., 2010). However, a single SNS usually consists of numerous topic-based VCs (Mirai and Nobuhiko, 2020), and audiences in different VCs would hold different knowledge and attitudes toward the same topic. The sudden expansion of intergenerational audiences in VCs would definitely further intensify these cognitive divergences (Nelissen and Van Den Bulck, 2017). When people disclose their personal information (including their thoughts, feelings and experiences) about a VC topic, their self-disclosure is very likely to be seen by others who have different cognitive preferences and value orientations with them, and then VC audiences’ risk of being involved in cyber ostracism or cyberbullying would be significantly increased (Forest and Wood, 2012; Kassner et al., 2012; Bauman et al., 2013; Changho and Namin, 2017). Cyber celebrities’ depression and suicide caused by their self-disclosure in VCs are the most intuitive responses to this phenomenon. To mitigate intergenerational VC audiences’ mental risks derived from their self-disclosure and to improve their psychological well-being, we introduced the concept of “social integration” into cyber society among non-acquaintance VCs.

The theory of social integration was put forward with the in-depth study of social ostracism and anti-ostracism (Albert, 1986). With the rapid spread of SNSs and ICTs, individuals in cyber society among topic-based VCs started to face the problem of cyber ostracism and cyber integration (Yang and Lee, 2018). Since social media usage and computer-mediated communication greatly reduce individuals’ time and money costs to adapt to the society they live in (including real society and cyber society) (Ewart and Snowden, 2012; Wei and Gao, 2016), many previous studies have indicated that social media is positively related to individuals’ social integration and well-being (including both subjective well-being and psychological well-being) (Brendler et al., 2013; Chan, 2015; Park et al., 2016; Chen and Li, 2017). However, cyber society, faced by intergenerational individuals with different value orientations and cognitive preferences, is quite different (Prensky, 2009; Wang et al., 2012; Nelissen and Van Den Bulck, 2017). As an important characterization of individuals’ harmonious social lives and stable psychological status in cyber society, a high social integration level would make VC audiences exhibit stronger psychological resilience toward the negative outcomes of their self-disclosure (Banoa et al., 2019). Existing studies generally pay attention to the psychological consequences (i.e., psychological well-being) of social integration (Chen and Li, 2017), but greatly ignore its psychological antecedents. Considering self-disclosure as the basic point of individuals’ social integration in topic-based VCs, this research explored VC audiences’ psychological factors throughout the entire social integration process and identified the relationships among these psychological factors. As Pang (2018) argued that social capital is positively linked to social integration, this research primarily focused on the psychological basis of VC audiences’ social integration from the perspective of their relational and cognitive social capital (Nahapiet and Ghoshal, 1998). Structural social capital in non-acquaintance VCs is usually unstable due to audiences’ relatively loose social interaction ties. In the context of topic-based VCs, we treated intergenerational VC audiences who frequently disclose themselves regarding certain topics as a whole. Our findings would have far-reaching significance in improving the social environment in the cyber-society among non-acquaintance VCs.

## Literature Review and Hypotheses

### Individuals’ Self-Disclosure in Virtual Communities and Intimacy

Non-acquaintance VCs in SNSs have increased individuals’ social participation opportunities and developed the social capital theory (Shah et al., 2001; Xiong et al., 2018). Individuals’ social relationships in VCs (including bridging relationships and bonding relationships) have gradually supplemented (or even substituted) the traditional, face-to-face social relationships people have in real society (Dijk, 1999; Ellison et al., 2014). Unlike acquaintance VCs (e.g., WeChat Groups), topic-based VCs in SNSs generally have the characteristics of virtuality, anonymity, and disinhibition (Prentice-Dunn and Rogers, 1982; McKenna et al., 2002; Chou et al., 2010). When people decide to disclose themselves in topic-based VCs, the weak social bonds and low moral restraint in this highly virtual setting greatly liberate them from the social barriers they might face otherwise (such as judgment, taboos, and social anxiety in real society) (Ben-Ze’ev, 2003; Hollenbaugh, 2010; Attrill and Jalil, 2011; Chen and Sharma, 2013). Hence, audiences’ willingness to disclose their personal thoughts, feelings, and experiences is greatly enhanced by the virtual environment in topic-based VCs.

According to the definition of self-disclosure (Cozby, 1973; Wheeless, 1978; Greene et al., 2006), VC audiences’ multiformed self-disclosure (including texts, pictures, audio, and video) should always be an interactive process that involves at least two subjects. Since individuals’ structural capital (i.e., social interaction ties) in topic-based VCs is relatively loose (Xiong et al., 2018), their objects of self-disclosure are usually unclear. The long-term absence of objects during individuals’ self-disclosure gradually leads them into self-isolation and then further affects their psychological status. However, interaction is always the essence of self-disclosure, and it would definitely require VC audiences to contact each other and eliminate their prejudice (Gao et al., 2015; Chen and Li, 2017; Cao et al., 2018). To shape positive attitudes and foster positive relationships through self-disclosure in topic-based VCs, VC audiences usually need to find targeted objects of their self-disclosure and build certain social bonds to compensate for the social structural hole in cyber society. Individuals’ intimacy needs to be formed through the interactive process of their self-disclosure (Perlman and Fehr, 1987; Prager, 1995; Park et al., 2011). Since VC audiences’ intimacy (i.e., individuals’ personal and subjective sense of connection) could reduce their self-alienation, advance their well-being (Ko and Kuo, 2009; Lee et al., 2010), and eventually help build their trust and reciprocal relationships in topic-based VCs (Baumeister and Leary, 1995; Laurenceau et al., 1998), we can consider intimacy as individuals’ perception of their close relationships with others in topic-based VCs.

In the context of this research, individuals’ self-disclosure cannot directly enhance their social integration. To promote audiences’ social integration in topic-based VCs, the information (such as personal thoughts, feelings, and experiences toward a VC topic) that individuals disclose needs to break their self-isolation and foster a certain degree of intimacy first. Therefore, individuals’ self-disclosure is an important antecedent of their intimate cultivation in topic-based VCs, and intimacy is the psychological foundation to accumulate their relational capital. Based on these analyses, we hypothesized the following.

H1: Individuals’ self-disclosure could positively affect their intimacy in VCs.

### Individuals’ Self-Disclosure in Virtual Communities and Cognitive Communion

Although the virtual environment in topic-based VCs has broadly promoted audiences’ self-disclosure (McKenna and Green, 2002; McKenna et al., 2002), audiences with different social needs usually selectively disclose their information (e.g., thoughts, feelings, and experiences toward VC topic) (Cho, 2007; Teo et al., 2015). Under the current circumstances, individuals’ self-disclosure in topic-based VCs will inevitably be interpreted by intergenerational audiences with different cognitive preferences and value orientations. Different knowledge and attitudes among VC audiences would easily lead them to engage in and be affected by cyber ostracism or even cyberbullying. Margalit (2010) emphasized that individuals’ sense of loneliness would be exacerbated when the information they disclose in VCs cannot attract others’ interest. To reduce the negative impact of VC audiences’ self-disclosure, we need to guide them to accumulate certain cognitive capital and to build a versatile semantic environment among the entire community based on their self-disclosure.

Cheng and Guo (2015) argued that cognitive social capital mostly centers on individuals’ social cognition, which contains their shared vision, goals and languages. In the context of this research, VC audiences’ cognitive capital in topic-based VCs not only contains their similar concepts regarding the VC and their common knowledge of the VC topic, but also includes their common representation, interpretation, and understanding of others’ self-disclosure. Obviously, VC audiences’ accumulation of cognitive capital could effectively lower their communication barriers, break their self-isolation, and thereby reduce their involvement in cyber conflict. As the subdimension of “held in common” from socially shared cognition (Brewer, 1991), cognitive communion could be defined as one’s perception of shared knowledge with others (Seongtaek et al., 2012). That means VC audiences who perceive higher cognitive communion with others would experience a higher level of like-mindedness (Brewer and Gardner, 1996).

Since individuals’ self-disclosure in VCs could always let others know them in the process of their information disclosure and feedback, it can definitely bring consistency into their own self-concept and others’ views of them (Attrill and Jalil, 2011). Thus, VC audiences’ perceptions of cognitive communion could weaken their ambiguousness toward others’ self-disclosure, strengthen their sense of similarity during this process (Riley and White, 2016), and guide them to accumulate certain cognitive capital in topic-based VCs. In this research, VC audiences’ self-disclosure is the chief factor in fostering their cognitive communion, which could reduce their psychological threshold and help them to accumulate cognitive capital in topic-based VCs. Hence, we proposed the following hypothesis.

H2: Individuals’ self-disclosure could positively affect their cognitive communion in VCs.

### Virtual Community Audiences’ Social Integration and Mental Health

Humans are social animals and always have the instinctive need to acquire social integration (Michael and Walter, 1981). To date, studies related to social integration have mostly focused on the field of pedagogy and social psychology. Pedagogical scholars hold that students’ social integration needs to be realized through their interaction with peers (Bossaert et al., 2011; Szumski and Karwowski, 2015). To seek social acceptance during the interactive process, students would develop their relationships and improve their social integration status, and a higher social integration level effectively increases students’ well-being in school life (Kong et al., 2015; Rubin et al., 2016). Meanwhile, social psychological scholars prefer to study the diversity of social integration based on individuals’ attitudes and ideologies (Verkuyten, 2007; Sarah, 2012), and they have explored the formation of social integration *via* social identity challenges and by redefining social boundaries (Chryssochoou, 2004; Moghaddam, 2008).

Regardless of the online or offline society, individuals’ shared interests, knowledge and shared language are always the basic foundations of their harmonious coexistence (Hu et al., 2017). Referring to the previous definition of social integration (Adachi, 2011; Goswami, 2012), social integration in the context of topic-based VCs should be considered “the common social concept that is shared by VC audiences with different cognitive preferences and value orientations.” To establish the common concept of cyber society in topic-based VCs, people need to accumulate certain relational capital and cognitive capital to ease their social barriers. Specifically, topic-based VC audiences’ self-disclosure could help them to cultivate intimacy with others. As the perception of close relationships with others, intimacy is the psychological premise for audiences to accumulate trust and reciprocal ties in topic-based VCs (Hsu et al., 2015). Meanwhile, as the perception of like-mindedness with others, VC audiences’ cognitive communion is also an important prerequisite to build common knowledge, create shared languages, and construct a versatile semantic environment among topic-based VCs (Stokburger-Sauer et al., 2012; Hu et al., 2017).

In topic-based VCs, audiences’ relational capital (i.e., trust and reciprocal relationships) and cognitive capital (i.e., common knowledge, shared language and versatile semantic environment) are both vital to their social participation (Hu et al., 2016), social identity formation (Cheng and Guo, 2015) and social network construction (Banoa et al., 2019). Since social participation, social identity and social networks are the three dimensions of social integration (Wei and Gao, 2016), VC audiences’ relational and cognitive capital accumulation would definitely promote their social integration level in cyber life and help them to obtain more social support (Lohbeck, 2020). Thus, individuals’ intimacy and cognitive communion derived from their self-disclosure are both key psychological antecedents for them to acquire social integration. Thus, we proposed the following hypotheses.

H3: Individuals’ intimacy could positively affect their degree of social integration in VCs.

H4: Individuals’ cognitive communion could positively affect their degree of social integration in VCs.

Furthermore, social support could positively affect individuals’ self-disclosure in VCs (Lin et al., 2021), and audiences who wish to obtain social support in topic-based VCs have to disclose themselves. The interactive progress of individuals’ self-disclosure would first help them cultivate their intimacy with the disclosing objects and then help them build certain relational capital in topic-based VCs (Chan and Lin, 2020). Hsiao and Chiou (2012) advocated that individuals’ cognitive capital in VCs should be realized on the basis of their relational capital accumulation (trust and reciprocity), and Xiong et al. (2018) also reinforced this conclusion. Individuals who disclosed their information in topic-based VCs could cultivate their intimacy with others, and then strengthen and consolidate their trust and reciprocal ties among VCs. Such relational capital could make VC audiences further experience their like-mindedness between themselves and others and perceive high cognitive communion (Hu et al., 2017). In short, individuals who want to perceive cognitive communication should first accumulate certain trust or reciprocal relationships in VCs based on their intimacy. Thus, we hypothesized the following.

H5: Individuals’ intimacy could positively affect their cognitive communion in VCs.

### Virtual Community Audiences’ Psychological Well-Being

Well-being refers to individuals’ perception and evaluation of their quality of life (Felfe and Yan, 2009), it contains individuals’ autonomy, self-acceptance, purpose in life, personal growth, environmental mastery, and positive relations with others (Ryff, 1989; Goswami, 2012; Chan, 2015). Because all these aspects of well-being mentioned above reflect the psychological view of individuals’ life experience, we can consider well-being as psychological well-being. In this research, audiences’ psychological well-being mostly centers on the judgment and evaluation of their own life in cyber society among VCs. According to the meta-analysis of Liu et al. (2019), individuals’ different social media uses have different impacts on their psychological well-being. The interactive process of self-disclosure, which has been greatly stimulated by the virtual environment in topic-based VCs, effectively fosters VC audiences’ intimacy with others. And VC audiences’ accumulation of relational capital and acquisition of social integration are subsequently promoted based on this psychological antecedent (i.e., intimacy) (Madge et al., 2009; Wei and Gao, 2016). Moreover, topic-based VC audiences’ social integration could in turn help them to obtain social support (including information support and emotion support) and enhance their perceived quality of cyber life (Ballantine and Stephenson, 2011; Hajli, 2014; Wen et al., 2016). Thus, we proposed the following hypothesis.

H6: Individuals’ acquired social integration could positively affect their psychological well-being in VCs.

Due to the unique properties of topic-based VCs, audiences’ structural social capital (i.e., social interaction ties) is usually unstable. However, the intimacy derived from the interaction process of individuals’ self-disclosure is conducive to their accumulation of relational capital (such as trust and reciprocal relationships), and the cognitive communion derived from individuals’ communication process during their self-disclosure contributes to their accumulation of cognitive capital (such as common knowledge, shared language and versatile semantic environment). Social capital is always positively associated with psychological well-being (Nieminen et al., 2010; Chan, 2015; Chan and Lin, 2020) because strong and weak ties can both help individuals reduce loneliness (Burke et al., 2010), circulate useful information (Granovetter, 1973) and provide social support (Leung and Lee, 2005). Thus, individuals’ intimacy and cognitive communion can both improve their psychological well-being in topic-based VCs. Based on the arguments above, we developed the following hypotheses.

H7: Individuals’ intimacy could positively affect their psychological well-being in VCs.

H8: Individuals’ cognitive communion could positively affect their psychological well-being in VCs.

In essence, social integration involves the concept of citizenship; it goes beyond basic kinship and unites individuals from different backgrounds (Banks, 2009). Therefore, social integration not only emphasizes individuals’ internal sense of belonging (e.g., peer support and subsequently enhanced intimacy) (Montoya et al., 2009), but also pays attention to the degree and intensity of individuals’ diverse accumulation of social capital (e.g., common social concept derived from cognitive communication) (Cheng et al., 2016). In the context of topic-based VCs, social integration enables VC audiences to reconstruct their social relationships according to their intimacy and establish their social engagement based on their cognitive communion (Madge et al., 2009; Sarah, 2012); in turn, their psychological well-being is improved as well. Thus, we posited that VC audiences’ social integration would significantly mediate the relationship between their intimacy and psychological well-being, and the relationship between their cognitive communion and psychological well-being. Based on these reviews, we hypothesized the following.

H9: Individuals’ acquired social integration is likely to mediate the relationship between their intimacy and psychological well-being in VCs.

H10: Individuals’ acquired social integration is likely to mediate the relationship between their cognitive communion and psychological well-being in VCs.

**FIGURE 1:**
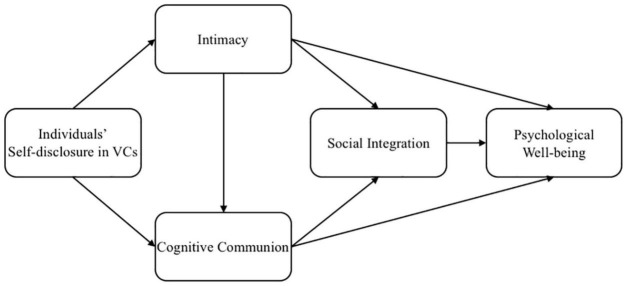
Research model.

Based on above hypotheses, the route model developed in this research is depicted in Figure 1.

## Materials and Methods

### Sample and Collection

To test these hypotheses, we conducted an online survey among participants whom were recruited from two different topic-based VCs among Microblog (i.e., Health Regimen VC and Star Chasing VC) to complete an online questionnaire. Health Regimen (HR) VC and Star Chasing (SC) VC are both relatively comprehensive and popular in Microblog. Although the target topics of audiences in HR VC and SC VC are not the same, both of these two VCs provide great public spaces for individuals to spontaneously get together and interact with each other. Since audiences in these two VCs are usually anonymous, their social relationships are relatively loose when initially joining. And their main purpose for interacting with each other is to talk about the VC topic (i.e., audiences in HR VC usually exchange information related to their health status and diet, while audiences in SC VC always prefer to talk about celebrities and entertainment gossip). The process of data collection lasted for nearly 2 months from March to May, 2021. During this time, 494 responses were collected from the Sina Microblog.

To ensure the diversity of intergenerational audiences in our sample, we just eliminated 22 invalid responses, including questionnaires that had the same answer for all items and questionnaires that were completed in less than 30 s. Our final sample consisted of 472 participants and represented a response rate of 95%. For these 472 participants, 208 were from HR VC and 264 were from SC VC. Their demographic statistics of the participants in this research are presented in Table 1. The data show that the age structure and education level of the participants in our survey correspond with the findings of the Sina Weibo Data Center (2021). In short, the age of VC audiences is relatively young (only 28.82% of them were born before 1985) and their education level is generally not high. Clearly, the user composition of topic-based VCs is the crucial foundation of the social conflict and social integration in cyber-society among Microblog.

**TABLE 1:** Demographic information.

Scales	Items	%
Gender	Male	42.37
	Female	57.63
Age	<18	16.53
	19–25	31.99
	26–35	22.67
	36–55	20.34
	>56	8.47
Educational level	Junior College and Below	41.13
	Bachelor’s degree	36.52
	M.Sc. or Ph.D. degree	22.35
Participation frequency	Almost everyday	63.59
	At least once a week	33.25
	At least once a month	2.37
	Hardly involved	0.79
Membership history	Less than 6 months	2.85
	About 1 year	26.16
	About 2 years	28.72
	More than 2 years	42.27

### Measures

Unless otherwise indicated, all variables are measured by participants’ responses to questions on a five-point Likert-type scale ranging from “strongly disagree” to “strongly agree”. To ensure the validation of the instrument, survey items were mostly adapted from scales developed and validated by previous studies.

To ensure the validation of the instrument, we mostly adapted survey items from scales developed and validated by previous studies. *Self-disclosure*: We adapted the three items measuring individuals’ self-disclosure in VCs from Park et al. (2011). The items are as follows: “I usually post my feelings or thoughts in the VC;” “I frequently share my personal experiences in the VC” and “How many posts do you create per month in the VC?”. *Intimacy*: We gauged intimacy with a modified Miller’s Social Intimacy Scale (MSIS: Miller and Lefcourt, 1982). The three items are as follows: “I like to communicate with people in the VC;” “I think people in the VC could understand me;” “People in the VC could encourage and support me when I’m unhappy.” *Cognitive communion*: The three items that measure cognitive communion are as follows (Brewer and Gardner, 1996): “I feel I share similar thoughts with people in the VC among Microblog;” “I feel I have common knowledge with people in the VC among Microblog” and “I feel I share the same perspective as others in the VC among Microblog.” *Social integration*: We measured social integration from its two main dimensions (i.e., social identity and social networks) proposed by Wei and Gao (2016). The items are as follows: “I identify with the VC;” “I have friends in the VC” and “Audiences in the VC can support me when I need it.” *Psychological well-being*: We adapted the three items measuring psychological well-being from the work of Diener et al. (2003). The items are as follows: “I am always optimistic when disclosing myself in the VC;” “Activities in the VC among Microblog are purposeful and meaningful to me;” “Some audiences in the VC among Microblog respect me.”

All the items were translated into Chinese by the authors. To ensure the accuracy of our translation, the questionnaire was verified and refined by one behavioral science professor and three senior doctoral students, who were quite familiar with and had conducted in-depth research on online behavior. The questionnaires were then pretested by students who often participated in Sina Microblog to ensure that the questionnaire items’ wordings was comprehensible, and that the translation had logical consistency and contextual relevance (Bock et al., 2005).

### Data Analysis

Structural equation modeling was used to test the models shown in Figure 1. The Mplus 8 software was employed for data analysis. We first tested the reliability and validity of the measurement model with a confirmatory factor analysis (CFA). After the reliability and validity were established, we examined the research model. Finally, we also tested the mediating effect using Mplus 8 software. To gauge the model fit, chi-square (X^1^) values, comparative fix index (CFI), Tucker-Lewis index (TLI), root mean square error of approximation (RMSEA), and standardized root mean square residual (SRMR) are reported. We adapted *X*^2^/d*f* < 5, CFI > 0.90, TLI > 0.90, RMSEA < 0.08 and SRMR < 0.05 as the criteria for model fitness (Zhang and Bartol, 2010).

### Measurement Model

As the three items that measured VC audiences’ self-disclosure used different measuring methods, we conducted a principal component factor analysis to test whether one factor could be extracted from these three items. Bartlett’s test of sphericity showed that the Kaiser–Meyer–Olkin (KMO) statistic of 0.701 was significant at a level of 0.001, indicating the appropriateness of using a principle component factor analysis on the data. And only one factor, which explained 74.5% of the variance, was extracted; item loadings were 0.784, 0.821, and 0.863, all above the required threshold of 0.5.

The construct reliability and validity were examined by CFA. We first assess the construct reliability with Cronbach’s alphas and composite reliability. As displayed in Table 2, the Cronbach’s alpha values ranged from 0.809 to 0.862, and the composite reliability ranged from 0.814 to 0.869, which were greater than 0.7, thus indicating a satisfactory level of reliability (Hatcher, 1994). Table 2 shows that the average variance extracted (AVE) from every construct is greater than 0.5. And Table 3 reports the loading of the items in our research model. As expected, all item loadings are significantly higher than 0.5 (Teo and King, 1996). This suggests good convergent validity of the constructions (Bagozzi and Yi, 1988).

**TABLE 2:** Composite reliability and average variance extracted (AVE).

Constructs	Cronbach’s alpha	Composite reliability	AVE
ISD in VCs	0.809	0.814	0.593
Social integration	0.827	0.838	0.634
Intimacy	0.821	0.827	0.614
Cognitive communion	0.836	0.825	0.611
Psychological well-being	0.862	0.869	0.690

**TABLE 3:** Measurement loading.

Constructs	Items	Standard loading
ISD in VCs	Q1	0.752
	Q2	0.772
	Q3	0.785
Social integration	Q4	0.806
	Q5	0.816
	Q6	0.766
Intimacy	Q7	0.771
	Q8	0.775
	Q9	0.805
Cognitive communion	Q10	0.756
	Q11	0.789
	Q12	0.799
Psychological well-being	Q13	0.859
	Q14	0.879
	Q15	0.748

We scrutinized the discriminant validity of our constructs was examined by comparing the square roots of AVE for individual constructs to the shared variances between constructs. As seen in Table 4, the square roots of the AVE for the individual constructs, the diagonal elements, are all greater than their corresponding correlation coefficients with other constructs. The results reveal that all variables in this study are positively correlated with each other.

**TABLE 4:** Correlations between latent constructs.

Constructs	IP	SI	IN	CC	PWB
IP in VCs (IP)	0.770				
Social Integration (SI)	0.563***	0.796			
Intimacy (IN)	0.598***	0.563***	0.784		
Cognitive Communion (CC)	0.567***	0.631***	0.586***	0.782	
Psychological Well-being (PWB)	0.584***	0.693***	0.601***	0.681***	0.831

The diagonal numbers are the square root of AVE. ***p < 0.01.

### Test for Common Method Variance

Data collected *via* a single self-report are susceptible to common method variance (CMV). In order to avoid CMV, we used a one-factor model approach to test for CMV (Liang et al., 2007). We connected all items to one latent variable and constructed a one-factor model, and we subsequently tested this model with confirmatory factor analysis (CFA). The overall fit indices of this one-factor model performed very poorly: RMSEA = 0.168, SRMR = 0.097, CFI = 0.691, TLI = 0.653, *X*^2^= 2462.989, d*f* = 152, *X*^2^/d*f* = 16.204. These indices are far beyond the acceptable range. Taken together, these results suggest that CMV did not pose a significant threat to the interpretation of our present findings (Harris and Mossholder, 1996).

## Results

### Structural Model

**FIGURE 2:**
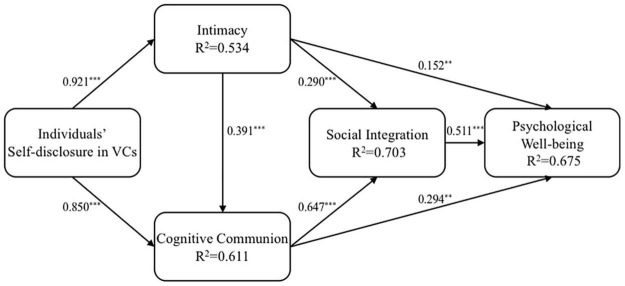
Model testing results. **p < 0.05; ***p < 0.01.

The overall fit indices of the research model are presented (as baseline model) in Table 5. As presented, all overall fit indices of the research model perform well; the CFI and TLI both perform above the threshold values. SRMR is less than 0.05. RMSEA is less than 0.08, close to 0.05. Figure 2 presents the overall structural model with path coefficients. The results of the SEM analyses for our research model support the majority of our hypotheses. First, hypotheses H1 and H3 are supported. The path coefficient of the relationship between individuals’ self-disclosure in VCs and their intimacy is 0.921 (*p* 0.001), and the path coefficient of intimacy and social integration is 0.290 (*p* 0.001). Meanwhile, hypotheses H2, H4, and H5 are supported. The path coefficient of relationship between individuals’ self-disclosure in VCs and cognitive communion is 0.850 (*p* 0.001), and the path coefficient of the relationship between cognitive communion and social integration is 0.647 (*p* 0.001). Furthermore, the direct relationship between intimacy and cognitive communion is also supported (H5: path coefficient = 0.391, *p* 0.001). These results indicate that individuals’ social integration in VCs needs to be realized on the basis of their intimacy and cognitive communion derived from self-disclosure. Second, hypotheses H6, H7, and H8 are also supported as social integration is positively associated with psychological well-being (H6: path coefficient = 0.62, *p* 0.001). Both intimacy and cognitive communion exert a positive effect on psychological well-being (H7: path coefficient = 0.152, *p* 0.005; H8: path coefficient = 0.294, *p* 0.005). These positive relationships indicate influences of social integration on individuals’ psychological well-being. The explained variances of intimacy, cognitive communion, social integration and psychological well-being are 53.4, 61.1, 70.3, and 67.5%, respectively, indicating that the model has a good predictive validity (Straub et al., 2004).

**TABLE 5:** Goodness of fit indices for the structural model.

Goodness of fit indices	Results	Desired levels
X^2^	250.102	Smaller
df	89	–
X^2^/df	2.810	<5
GFI	0.962	>0.9
TLI	0.952	>0.9
SRMR	0.033	<0.05
RMSEA	0.047	<0.08

### Mediation Effect

As studies indicate that bootstrapping is the most powerful and reasonable method for obtaining confidence limits for specific indirect effects under most conditions (Preacher and Hayes, 2008), the bootstrapping method is used to test the mediation effects.

Table 6 suggests that the indirect effect of intimacy on psychological well-being through social integration is 95% likely to range from 0.318 to 0.628, and the estimated effect is 0.461, which lies between these two values. Zero does not occur between the lower and upper limits and the *p* value is smaller than 0.001. Therefore, we conclude that the indirect effect is significant and that social integration partially mediates the relation between intimacy and psychological well-being. Moreover, the indirect effect accounts for 54.1% of the total effect. Thus, H9 is supported.

**TABLE 6:** Mediating effect of social integration between intimacy and psychological well-being.

	PWB		SI		Estimate	S.E.	Bootstrapping BC 95% CI	
	Estimate	S.E.	Estimate	S.E.			Lower limit	Upper limit
IN	0.869***	0.027	0.391***	0.100				
PWB			0.530***	0.104				
R^2^	0.407		0.563					
Indirect effect					0.461***	0.094	0.318	0.628
Direct effect					0.391***	0.099	0.218	0.546
Total effect					0.852***	0.022	0.730	0.805

***p < 0.01.

Table 7 suggests that the indirect effect of cognitive communion on psychological well-being through social integration is 95% likely to range from 0.193 to 0.562, and the estimated effect is 0.405, which lies between these two values. Zero does not occur between the lower and upper limits and the *p* value is smaller than 0.001. Therefore, we conclude that the indirect effect is significant and that social integration partially mediates the relation between cognitive communion and psychological well-being. Moreover, the indirect effect accounts for 45.5% of the total effect. Thus, H10 is supported.

**TABLE 7:** Mediating effect of social integration between cognitive communion and psychological well-being.

	PWB		SI		Estimate	S.E.	Bootstrapping BC 95% CI	
	Estimate	S.E.	Estimate	S.E.			Lower limit	Upper limit
CC	0.910***	0.021	0.485***	0.103				
PWB			0.446***	0.153				
R^2^	0.499		0.591					
Indirect effect					0.405***	0.134	0.193	0.562
Direct effect					0.485***	0.143	0.253	0.615
Total effect					0.891***	0.021	0.656	0.725

***p < 0.01.

Table 8 presents the comparison of the indirect effects of intimacy and cognitive communion on psychological well-being through social integration. An examination of the contrast of these two indirect effects (C1) indicates that the indirect effect of cognitive communion on psychological well-being is significantly greater than the indirect effect of intimacy on psychological well-being, with a BC 95% C between −0.429 and −0.002 being observed.

**TABLE 8:** Indirect effects of intimacy and cognitive communion on psychological well-being through social integration.

	Estimate	S.E.	Bootstrapping BC 95% CI	
			Lower limit	Upper limit
Intimacy	0.171	0.075	0.069	0.311
Cognitive communion	0.364	0.108	0.211	0.578
C1	−0.194	0.125	−0.429	−0.002

*C1, contrast of the two indirect effects, 1000 bootstrap samples.*

## Discussion

Cyber society among VCs has greatly developed and extended our real-society (Erin and Amber, 2015) under the circumstance of post COVID-19. Although the topic-based VCs on Microblog have recently been full of intergenerational audiences, most of their users are still relatively young. In our sample, 48.52% of the participants were born after 1995, and 22.67% of the participants were born from 1985 to 1995. These VC audiences are usually more extroverted and perceptual. Nearly 28.81% of VC audiences were born before 1985 in our sample, and these audiences are usually more conservative and persistent. At the same time, the 22.35% highly educated audiences in our sample are more likely to be rational and comprehensive than others. Obviously, intergenerational audiences’ different cognitive preferences and value orientations are widely divergent. To develop and maintain VC audiences’ social function, and construct a multivalued social environment in cyber society, we need to avoid the frequent cyber ostracism (and even cyberbullying) caused by VC audiences’ self-disclosure, and guide them to acquire social integration in topic-based VCs. Recent studies related to online self-disclosure have mostly explored its impact on social relationships (Lee et al., 2021), but have greatly ignored VC audiences’ psychological perceptions of it. To alleviate the negative impact of VC audiences’ self-disclosure (i.e., mental damage and value conflict caused by self-disclosure), we introduced the theory of social integration into cyber society among topic-based VCs.

Our model explored and identified the psychological antecedents and consequences of VC audiences’ social integration. Taking individuals’ self-disclosure in VCs as an application or system usage (Klimoski and Mohammed, 1994), it can help individuals produce meaning and emotions together (Forlizzi and Battarbee, 2004), and then cultivate intimacy and cognitive communion with others in VCs. VC audiences’ intimacy and cognitive communion can enable them to acquire social integration. Meanwhile, as the psychological consequences of VC audiences’ social integration studied in this research, psychological well-being could partially avoid the negative impact of individuals’ self-disclosure in VCs. From the perspective of audiences, individuals who want to improve their psychological well-being need to enhance their social integration level by cultivating intimacy and cognitive communion with others when disclosing themselves in topic-based VCs. From the standpoint of VCs, intimacy and cognitive communion fostered in the process of individuals’ self-disclosure could drive them to accumulate social capital (including both relational capital and cognitive capital) and enhance their social integration, which is positively tied to their VC commitment and stickiness. Our findings clarify that audiences’ self-disclosure cannot directly enhance their social integration and improve their psychological well-being in topic-based VCs.

The theoretical model constructed in this research not only reduced the negative effects of individuals’ self-disclosure in topic-based VCs, but also identified individuals’ psychological antecedents and consequences of their social integration in VCs. In terms of theoretical contributions, this research has broadened existing social integration studies, applied it to the scene of cyber society among topic-based VCs, and explored the psychological antecedents and consequences of VC audiences’ social integration. In addition, this research has several significant implications for practice. Our results indicate that individuals who wish to acquire social integration and improve their psychological well-being need to primarily disclose their own information (e.g., personal thoughts, feelings, and experiences) that could cultivate intimacy and cognitive communion with others. It is only through intimacy and cognitive communion that VC audiences can accumulate relational and cognitive capital and obtain social and emotional support in cyber society they live in Pang (2018). Our findings are important for the future popularization of online learning and telecommuting scenarios.

Moreover, currently customized services provided by VCs among SNSs meet the needs of their audiences well through big data algorithms (Marianne and Christian, 2016). However, they have also intensified VC audiences’ difficulty in engaging in a wider range of interactions and strengthened their ideological isolation. VC audiences’ psychological antecedents and consequences of social integration explored in this research would weaken the negative impact of current big data algorithms to a certain extent.

## Conclusion

Since intergenerational audiences’ social lives in non-acquaintance (i.e., topic-based) VCs have aroused great attention, we aimed to explore and identify the psychological antecedents and consequences of individuals’ social integration in cyber society. Considering previous studies about online self-disclosure (Cho, 2007; Erin and Amber, 2015; Lee et al., 2021; Lin et al., 2021) and offline social integration (Wei and Gao, 2016; Wen et al., 2016; Pang, 2018), we have extended the research of individuals’ social integration in cyber society to a more detailed level. Our results show that individuals’ intimacy and cognitive communion could be derived from their self-disclosure in topic-based VCs, and VC audiences’ intimacy and cognitive communion could enhance their social integration. Thus, both intimacy and cognitive communion are psychological antecedents of VC audiences’ social integration. Meanwhile, as the chief psychological consequence of their social integration, VC audiences’ psychological well-being is positively related to their intimacy and cognitive communion. Further, social integration plays a significant mediating role between intimacy and psychological well-being, and between cognitive communion and psychological well-being. Overall, VC audiences who wish to improve their psychological well-being could acquire social integration in cyber society first. In addition, VC audiences’ intimacy could positively affect their cognitive communion. Concentrating on social integration in cyber society may help individuals to deeply understand the intrinsic motivation of their social integration in topic-based VCs and then guide them to cultivate a high-quality online (and even offline) social life.

## Limitations and Future Research

The limitations in this study are as follows. First, we mentioned the negative impacts of intergenerational audiences’ self-disclosure in topic-based VCs as a realistic background, i.e., individuals’ self-disclosure based on their great cognitive divergence would easily result in cyber tsunami. However, the specific path of this phenomenon has not been deeply analyzed in this research. To avoid this phenomenon, future studies should not only explore the negative impact of VC audiences’ self-disclosure, but also further clarify its formation path. Second, we conducted our research in two topic-based VCs among Microblog wherein audiences usually know very little about each other. The psychological antecedents and consequences of VC audiences’ social integration identified in this research might not totally apply to all types of VCs. Thus, future research could explore the driving and influencing mechanisms of social integration in acquaintance VCs, such as a WeChat Group. In addition, we examined social integration among topic-based VC audiences to improve cyber society in topic-based VCs. Considering the digital divide, future studies could focus on the social integration mechanisms of diverse audience groups in topic-based VCs (such as digital natives and digital immigrants) and investigate the differences among them.

## Data Availability Statement

The original contributions presented in the study are included in the article/Supplementary Material, further inquiries can be directed to the corresponding author.

## Author Contributions

YZ: article theme and theoretical model conception and full text writing. ZC: research direction and thesis writing guidance. YP: questionnaire preparation and data collection. YX: article revision. All authors contributed to the article and approved the submitted version.

## Conflict of Interest

The authors declare that the research was conducted in the absence of any commercial or financial relationships that could be construed as a potential conflict of interest.

## Publisher’s Note

All claims expressed in this article are solely those of the authors and do not necessarily represent those of their affiliated organizations, or those of the publisher, the editors and the reviewers. Any product that may be evaluated in this article, or claim that may be made by its manufacturer, is not guaranteed or endorsed by the publisher.
