# Supplementary material for: Psychological Antecedents and Consequences of Social Integration Based on Self-Disclosure in Virtual Communities: Empirical Evidence From Sina Microblog
Source: Front Psychol. 2022 Feb 16;13:829327. doi: 10.3389/fpsyg.2022.829327 (PMC8888404; doi:10.3389/fpsyg.2022.829327)
Supplement: Supplementary file 1 [file Table_1.DOCX]

**Questionnaire Item List**

| Item | Questionnaire |
| --- | --- |
| Self-disclosure | I usually post my feelings or thoughts in the VC. |
| (Park et al., 2011) | I frequently share my personal experiences in the VC. |
|  | How many posts do you create per month in the VC. |
| Intimacy | I like to communicate with people in the VC. |
| (MSIS: [Miller & Lefcourt, 1982](#Miller1982)) | I think people in the VC could understand me. |
|  | When I’m unhappy, people in the VC could encourage and support me. |
| Cognitive communion | I felt I shared similar thoughts with others in the VC among Microblog. |
| ([Brewer, Gardner, 1996](#Brewer1996)) | I felt I have common knowledge with others in the VC among Microblog. |
|  | I felt I shared the same perspective as others in the VC among Microblog. |
| Social integration | I identify with the VC. |
| ([Wei & Gao, 2016)](#Wei2016) | I have friends in the VC. |
|  | Audiences in the VC could support me when I need. |
| Psychological Well-being | I am always optimistic when disclosing myself in the VC. |
| ([Diener, Oishi, & Lucas, 2003)](#Diener2003) | Activities in this VC among Microblog are purpose and meaningful to me. |
|  | Some audiences in the VC among Microblog respect me. |

Park, N., Jin, B., Annie, Jin., 2011. Effects of self-disclosure on relational intimacy in Facebook. Computers in Human Behavior, 27 (5), 1974-1983. [https://doi.org/10.1016/j.chb.2011.05.004.](https://doi.org/10.1016/j.chb.2011.05.004)

Miller, R. S., Lefcourt, H. M., 1982. The assessment of social intimacy. Journal of Personality Assessment, 46(5), 514–518. [https://doi.org/10.1207/s15327752jpa4605_12.](https://doi.org/10.1207/s15327752jpa4605_12)

Brewer, M. B., & Gardner, W., 1996. Who is this ‘‘we’’?: Levels of collective identity and self-representations. Journal of Personality and Social Psychology, 71(1), 83–93. [https://doi.org/10.1037/0022-3514.71.1.83.](https://doi.apa.org/doi/10.1037/0022-3514.71.1.83)

Wei L., Guo F., 2016. Social media, social integration and subjective well-being among new urban migrants in China. Telematics and Informatics, 34 (3), 786-796. <http://dx.doi.org/10.1016/j.tele.2016.05.017>

Diener, E., Oishi, S., & Lucas, R. E., 2003. Personality, culture, and subjective well-being: Emotional and cognitive evaluations of life. Ann. Rev. of Psy., 54(1), 403–425. <https://doi.org/10.1146/annurev.psych.54.101601.145056>.
